# Polyphenol Effects on Cholesterol Metabolism via Bile Acid Biosynthesis, CYP7A1: A Review

**DOI:** 10.3390/nu11112588

**Published:** 2019-10-28

**Authors:** Karen F. Chambers, Priscilla E. Day, Hassan T. Aboufarrag, Paul A. Kroon

**Affiliations:** Food & Health, Quadram Institute Bioscience, Norwich Research Park, Norwich, Norfolk NR4 7UA, UK; karen.chambers1982@hotmail.co.uk (K.F.C.); priscilla.day@quadram.ac.uk (P.E.D.); Hassan.Aboufarrag@quadram.ac.uk (H.T.A.)

**Keywords:** atherosclerosis, reverse cholesterol transport, diurnal rhythms, microRNA, ASBT, flavonoid, phenolic acid, catechin, anthocyanin

## Abstract

Atherosclerosis, the main contributor to coronary heart disease, is characterised by an accumulation of lipids such as cholesterol in the arterial wall. Reverse cholesterol transport (RCT) reduces cholesterol via its conversion into bile acids (BAs). During RCT in non-hepatic peripheral tissues, cholesterol is transferred to high-density lipoprotein (HDL) particles and returned to the liver for conversion into BAs predominantly via the rate-limiting enzyme, cholesterol 7 α-hydroxylase (CYP7A1). Numerous reports have described that polyphenol induced increases in BA excretion and corresponding reductions in total and LDL cholesterol in animal and in-vitro studies, but the process whereby this occurs has not been extensively reviewed. There are three main mechanisms by which BA excretion can be augmented: (1) increased expression of CYP7A1; (2) reduced expression of intestinal BA transporters; and (3) changes in the gut microbiota. Here we summarise the BA metabolic pathways focusing on CYP7A1, how its gene is regulated via transcription factors, diurnal rhythms, and microRNAs. Importantly, we will address the following questions: (1) Can polyphenols enhance BA secretion by modulating the CYP7A1 biosynthetic pathway? (2) Can polyphenols alter the BA pool via changes in the gut microbiota? (3) Which polyphenols are the most promising candidates for future research? We conclude that while in rodents some polyphenols induce CYP7A1 expression predominantly by the LXRα pathway, in human cells, this may occur through FXR, NF-KB, and ERK signalling. Additionally, gut microbiota is important for the de-conjugation and excretion of BAs. Puerarin, resveratrol, and quercetin are promising candidates for further research in this area.

## 1. Introduction

Atherosclerosis, the main contributor to coronary heart disease, is characterised by an accumulation of lipids in the arterial wall [1]. Polyphenols have been shown to confer beneficial effects against cardio-metabolic diseases. Among the mechanisms proposed for their beneficial effects is the alteration of bile acid metabolism. Here, we give a comprehensive review of research on the molecular mechanisms through which polyphenols exert their beneficial effects focusing on CYP7A1 and bile acid metabolism. The key points raised in this review include:

1. Polyphenols have been shown to have a wide range of beneficial effects, of note red wine, rich in flavonoids, phenolic polymers and resveratrol are promising as possible targets for further investigation.
2. As well as giving a state-of- the-art review on the mechanisms through which polyphenols exert their beneficial effects focussing on CYP7A1 and bile acid metabolism, critical points that will be of benefit to clinical nutritionists, academic experts in the area of bioactive food compounds, and possible stakeholders have also been raised in this review namely;
  - While polyphenols do have effects on bile acid metabolism, it should be born in mind that species differences, time of cull, dose, and length of treatment can also affect the results and as such, may lead to discrepancies between studies. As such, there is a need to standardise animal studies, ensuring that these factors are correctly reported.
  - Polyphenols confer their beneficial effects partly through altering the microbiome, thus they could have useful prebiotic-like functions.
  - There is a lack of data on the effects conferred by the parent compound versus those of their metabolic products, and future studies should aim to determine the effects of both the parent compounds and their metabolites, with particular emphasis on the gut microbiota.
  - Further studies are required in humans to substantiate the mechanisms proposed using animal and cell culture models.

### 1.1. Reverse Cholesterol Transport

**Figure 1:**
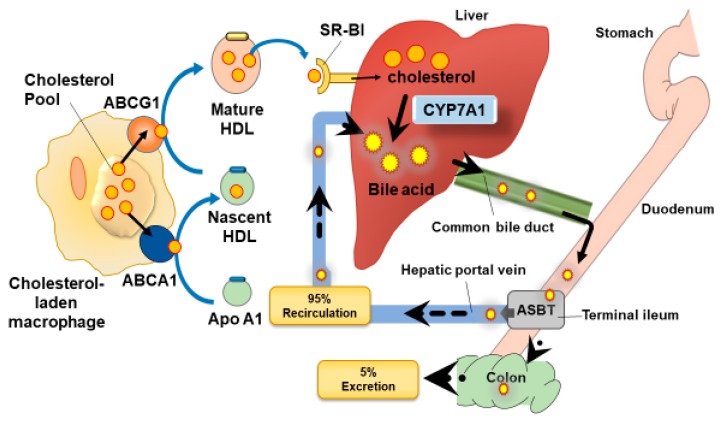
The enterohepatic re-circulation of bile acids via reverse cholesterol transport. Cholesterol laden macrophages in the arterial wall deliver cholesterol via the ABCA1 transporter to lipid free apoA-I, preventing foam cell formation and also forming nascent HDL particles. Further lipidation of the nascent HDLs occurs via ABCG1. Cholesterol is delivered to the liver from mature HDL particles via specific HDL cholesterol efflux (CE) uptake by a scavenger receptor class B type I (SR-BI). In the liver, cholesterol is converted into BAs predominantly by the CYP7A1 neutral (classic) pathway. The BAs travel via the bile duct to the intestine, where they are de-conjugated via the bacteria and excreted or re-circulated (95%), usually in their conjugated form via passive diffusion or via active transport via the apical sodium dependent BA transporter (ASBT).

The reverse cholesterol transport (RCT) pathway removes excess cholesterol from the peripheral system including from lipid laden macrophages, thus preventing foam cell accumulation during atherosclerosis development. In this process, cholesterol is returned to the liver where it is converted into BAs predominantly via the cytochrome P450 enzyme, cholesterol 7 α-hydroxylase (CYP7A1), for subsequent excretion into the faeces (Figure 1).

**Figure 2:**
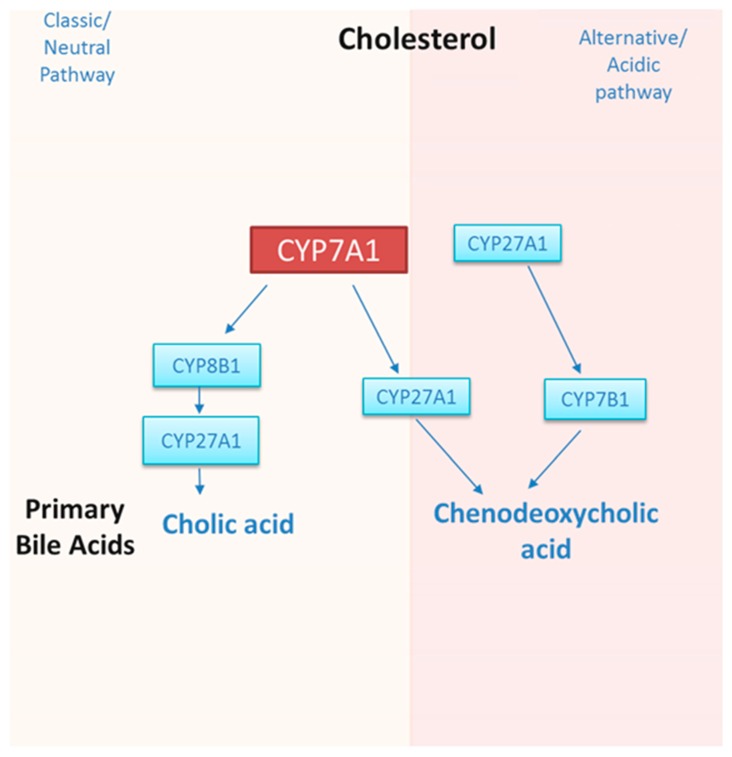
A summary of the main enzymes involved in the classic and alternative bile acid biosynthesis pathways. The classic pathway is controlled by the rate limiting enzyme CYP7A1 and the alternative pathway is controlled by CYP27A1; both pathways culminate in the production of cholic acid (CA) and chenodexoycholic acid (CDCA), the ratio of which depends on the activity of CYP8B1. Briefly, bile-acid biosynthesis begins with the modification of the ring structure of cholesterol, which involves oxidation and shortening of the side chain [2]. In the classic pathway, cholesterol is converted into 7 α-hydroxycholesterol by CYP7A1 and in subsequent steps, cytochrome P450 Family 8 Subfamily B Member 1a (CYP8B1) and sterol 27-hydroxylase (CYP27A1) are required for the synthesis of cholic acid (CA). Without CYP8B1, the product is chenodeoxycholic acid (CDCA), which is formed via the activity of CYP27A1 alone. The acidic pathway (or alternative pathway) is initiated by CYP27A1 and relies on 25-hydroxycholesterol 7-alpha-hydroxylase (CYP7B1) to produce CDCA.

In humans, approximately 500 mg/day of cholesterol is converted into bile acids (Bas) in the liver and lost in the faeces [2]. Two pathways, namely the neutral (classic) and the alternative (acidic) pathways, are involved in cholesterol metabolism, with CYP7A1 being the rate-limiting enzyme in the former, and CYP7A1 being responsible for cholesterol metabolism in the latter. Both pathways result in the formation of the primary BAs chenodeoxycholic acid (CDCA) and cholic acid (CA), the neutral pathway being the most predominant [3]. The acidic pathway only contributes 9% and 25% of total BAs in humans and mice, respectively [4]. The biochemistry of BA biosynthesis is reviewed elsewhere [2,5] and is summarised in Figure 2.

Before excretion into bile, primary BAs are conjugated with amino acids glycine and to a lesser degree taurine in humans [6]. In rodents, taurine is almost exclusively used for conjugation [6]. This increases solubility, minimises passive absorption, and makes the BAs resistant to cleavage by pancreatic carboxypeptidase. BAs are secreted from the gallbladder via the bile duct into the intestine and can be metabolised into their respective secondary BAs via the gut microbiota, which are also responsible for de-conjugation. Conjugated BAs are actively exported from the liver via the bile salt export pump (BSEP)/ABCB11, which in humans preferentially transports conjugated BAs, but can also transport unconjugated BAs [7]. The main function of BAs is to facilitate digestion in the gut and their synthesis inadvertently regulates lipid concentrations. A list of all known BAs and their abbreviations is given in Supplementary Table S1. BAs are reabsorbed in the intestine by either passive diffusion or by active transport, which occurs via the apical sodium BA transporter (ASBT) in the terminal portion of the ileum [8]. Once reabsorbed, BAs travel through the hepatic portal vein and are returned to the liver via specific transporters, with approximately 95% of BAs being recirculated back to the gallbladder in a process termed enterohepatic circulation, and the rest is excreted in the faeces (Figure 1). Typically, cholesterol synthesis equals its secretion as BAs; however, this equilibrium can be disturbed during disease states or by diet [9].

### 1.2. Regulation of *CYP7A1* by Dietary Cholesterol, Circadian Rhythm, Transcription Factors, and microRNAs

Diet can regulate BA synthesis, particularly the intake of a cholesterol rich diet. In wild-type mice, feeding a high cholesterol diet stimulated BA synthesis by activating LXRα to induce *CYP7A1* gene transcription [10,11,12]. Cholesterol treatment of HepG2 cells also moderately induces *CYP7A1* gene expression [13]. In contrast, another study using HepG2 cells, cholesterol dose dependently decreased *CYP7A1* expression [14], which is supported by a mouse study [15]. Although it has been suggested that mice have a different response to cholesterol when compared to humans, here, comparable effects were observed on BA pool size, faecal BA excretion, and plasma cholesterol levels between humans, transgenic mice, and wild-type CYP7A1 mice. This suggests that another mechanism other than LXR signalling alters BAs on a high cholesterol diet, perhaps the microbiota [16]. There are also differences between rodents and humans, for example LXRα-CYP7A1 signalling, is redundant in humans due to the lack of LXRE in the human *CYP7A1* gene promoter, but this pathway is well known in rodent models. Nevertheless, FXR signalling is homologous between mice and humans and is a well-studied mechanism of *CYP7A1* control. It is logical that cholesterol, the substrate for *CYP7A1*, would increase CYP7A1 levels, but the data published to date are equivocal and may depend on the presence of a functional LXRα receptor.

One key mechanism is the circadian regulation of CYP7A1. *Cyp7a1* levels in mice have been shown to peak mid-morning to noon, although this is also influenced by the fed and non-fed state [17]. This is particularly problematic when animals treated with different diets are culled at different times over the day as this would dramatically alter the results and lead to erroneous interpretation [17]. The circadian control of *Cyp7a1*, along with other clock associated genes, has also been shown to parallel changes in triglyceride and total cholesterol [18]. A summary of circadian genes involved in the regulation of *Cyp7a1* is shown in Table 1. *Cyp7a1* is also regulated by microRNAs, which are non-coding RNAs of 15–25 bases that bind to complementary sequences in the 3′-UTR regions of target mRNA to repress translation. The effects and targets of microRNAs on *Cyp7a1* and cholesterol metabolism are also summarised in Table 1. The concept that diet can regulate BA synthesis is not a new one, in fact fibre is well known to regulate BA synthesis [19,20,21,22,23], however, the idea that bio-actives within food, particularly polyphenols, can increase CYP7A1 without increased food consumption is an exciting field and will be considered in the following sections.

## 2. Current Status of Knowledge: Polyphenols That Regulate Bile Acid Synthesis and CYP7A1

Numerous studies have assessed the effects of polyphenols on BA excretion and CYP7A1 expression and the mechanisms by which they alter BA excretion and CYP7A1expression have been summarised in Supplementary Table S2; these were mostly obtained from rodent studies showing a depletion of hepatic cholesterol when fed polyphenols, which in some instances is linked to an increase in CYP7A1 activity and LXRα signalling. Polyphenols are subdivided into different categories: flavonoids, isoflavonoids, lignans, stilbenes, phenolic acids, and phenolic polymers (Supplementary Figure S1). It is worth noting that all types of polyphenols are only partially bioavailable and subject to both phase-2 conjugation and catabolism by the gut microbiota, and the reader is referred to some excellent reviews of the bioavailability and metabolism of polyphenols and how these processes can affect their biological activities [48,49,50,51,52]. Currently, evidence for whether the known effects of polyphenols on BA metabolism and CYP7A1 are mediated by the parent polyphenols or their metabolites, or a combination of both is limited; this is an important area for future research. There are multiple pathways through which polyphenols confer their beneficial effects including those involved in inflammation. These have been substantially reviewed elsewhere [53,54] Another biological activity of polyphenols is as antioxidant molecules that have the potential to mop up free radicals. Although this has been somewhat challenging to prove unequivocally in humans, it is notable that there is a significant body of evidence to show that consumption of specific polyphenols such as those in olive oil can effectively reduce the levels of oxidised LDL [55]. In the following sections, we review the effects of each sub-group of polyphenols on CYP7A1 expression, BA metabolism/excretion, and comment on the mechanism of action. We then further discuss the emerging research that polyphenols can mediate cholesterol efflux and microbial changes in the gut, subsequently modulating bile acid excretion.

### 2.1. Flavonoids

Flavonoids are water soluble polyphenolic molecules that consist of six major subgroups: flavanols (catechins), anthocyanins, flavonols, flavanones, and flavones (see Supplementary Figure S1). The effects of flavonoids on the HDL delivery of cholesterol to the liver via RCT have been recently reviewed [56], however, the effects of polyphenols on bile acid biosynthesis and efflux was not described.

#### 2.1.1. Flavanols

Flavanol-rich green tea has been shown to increase BA excretion, however, the link to CYP7A1 is tentative. For example, Lung Chen tea has been shown to reduce serum cholesterol and increase faecal BA excretion in hypercholesteraemic rats, without altering Cyp7a1 enzyme activity [57]. *Cyp7a1* gene expression was not changed in C57BL mice fed green tea extract (GTE) over six weeks [15]. Despite this, there was an increase in circulating BAs and excreted faecal cholesterol, although this was only apparent once normalised to the amount of faeces excreted per day as the mice on the GTE produced more faeces overall [15]. Interestingly, in the same study, GTE dramatically increased *Cyp27a1* mRNA expression in mice fed a high cholesterol diet, but not in mice fed a normal chow diet; indicating a shift from the neutral pathway to the acidic pathway only in the presence of high cholesterol [15]. Several studies have shown an increase in *Cyp7a1* expression in response to catechin treatment. For example, in rats, epigallocatechin gallate (EGCG) decreased bile acid-independent bile flow but not excretion, whilst increasing *Cyp7a1* mRNA expression and increasing circulating BAs [58]. EGCG increased Cyp7a1 protein levels in C57BL/6 mouse livers, however, BAs were not measured [59]. Finally, an in vitro study using HepG2 cells has shown that catechins increase *CYP7A1*mRNA levels with epicatechin gallate showing the greatest fold induction [60]. In contrast, EGCG is also known to be an activator of FXR signalling [61], which would in turn supress *CYP7A1*expression. Studies in our lab confirm a reduction of *CYP7A1* gene expression in human liver HepG2 cells treated with 5 µM EGCG in the absence of serum, which is consistent with the hypothesis that EGCG is an activator of farnesoid X receptor (FXR) signalling (unpublished data). Interestingly, in the presence of chenodeoxycholic acid, another FXR inhibitor, EGCG acts as a suppressor of FXR signalling in vitro [61]. *CYP7A1* levels were not measured, but it would be fascinating to explore the role of EGCG on CYP7A1expression in the presence and absence of FXR inhibitors.

#### 2.1.2. Anthocyanins

There are very limited data on the effects of anthocyanins on BAs. In one study, red pericarp glutinous rice, rich in anthocyanins, induced *Cyp7a1* expression when fed to hypercholesterolemic C57BL/6 mice when compared to brown rice [62]. A decrease in hepatic cholesterol was also observed although faecal cholesterol or BAs were not measured [62]. Lingonberries, which contain high levels of anthocyanins, have been shown to increase *Cyp7a1* expression and decrease atherosclerotic plaque and triglyceride concentration in *ApoE-/-* mice on a high fat diet [63]. The relative faecal abundance of the bacterial genera *Bacteroides*, *Parabacteroides*, and *Clostridium* were also increased [63]. In type-2 diabetic mice, Buckwheat sprouts, which contain cyanidin 3-O-glucoside (C-3-G) [64] and C-3-G alone, increased faecal BAs and *Cyp7a1* mRNA expression in the liver whilst correspondingly decreasing serum cholesterol, liver cholesterol, and triglycerides and consequently reduced atherosclerosis [65,66]. However, buckwheat protein has also been investigated as the active cholesterol lowering component [67]. High doses of polyphenol-rich Lonicera caerulea berry extract, which contains mainly anthocyanins, significantly upregulated *Cyp7a1* gene expression, reduced the expression of SREBP-1C, SREBP2, miR-33, and miR-122 and caused a reduction in cholesterol, LDL, and triglyceride in Sprague Dawley rats [68]. To our knowledge, all the published anthocyanin data that have examined changes in BA levels or *Cyp7a1* expression have utilised mouse models. However, human cells may respond differently, as in mouse primary hepatocytes, C-3-G treatment increased *Cyp7a1* expression in an LXRα dependent way [66]. Indeed, using HepG2 cells, we showed that C-3-G does not increase *CYP7A1* mRNA expression (data unpublished), perhaps because human cells do not possess functional LXRα.

#### 2.1.3. Flavonols

Quercetin, a flavonol found in capers, has been shown to elevate hepatic Cyp7a1 as well as LXRα at both mRNA and protein levels in male Wistar rats along with increased secretion of BAs and total hepatic BAs [69]. Importantly, quercetin also increased the expression of hepatic ATP binding cassette transporter G1 (ABCG1) mRNA and protein expression, indicating that quercetin may be involved in the regulation of hepatic cholesterol efflux [69]. Black bean seed coat extract predominantly containing quercetin 3-O-glucoside significantly stimulated the expression of Cyp7a1 protein in the liver and faecal BAs in C57BL mice [70]. A combination of quercetin and leucodelphinidin or quercetin with Banyan tree, derived leucopelargonin and leucocyanin significantly increased hepatic and faecal BAs in hypercholesteraemic rats and correspondingly reduced L-LDL cholesterol with the former also increasing HDL [71,72]. Overall, there is some evidence from animal studies that quercetin alone or in combination with other flavonoids can induce BA excretion via induction of CYP7A1. Other flavonols such as kaempferol have been shown to increase hepatic CYP7A1, faecal cholesterol, and BAs through mechanisms that may involve its binding to LXRα [73]. Human interventions and in vitro studies are still required to examine the role of quercetin and kaempferol on Cyp7a1 and lipid metabolism.

#### 2.1.4. Flavanones, Flavones, and Isoflavones

Naringin (a flavanone) occurs naturally in citrus fruits, especially in grapefruit, where it is responsible for the fruit’s bitter taste. Naringin, has been shown to induce LDL-receptor and *CYP7A1* expression in HepG2 cells through the NF-κB and ERK signalling pathway as well as through PPARy, which occurs in a dose-dependent manor [74,75]. Its aglycone naringenin has been shown to be a partial agonist of LXRα in cells transfected with a reporter construct [74]. In contrast, using computer modelling of tetrahydro-flavanones (cryptochinones A–D), it was shown that flavanones may behave as FXR agonists to decrease *CYP7A1* mRNA expression [76]. The only one study that investigated the effects of flavones from a leaf extract of Xanthosoma sagittifolium showed no effects on total Bas, but reduced secondary BAs in rats [77,78].

When administered to mice and rats, an isoflavonoid puerarin (from arrowroot) increases hepatic *Cyp7a1* expression and suppresses serum and hepatic cholesterol, although in HepG2 cells, no changes were observed [79,80,81]. Additionally, Xuezhikang, an isoflavone-rich extract of red-yeast-rice and *Erythrina lysistemon* also rich in isoflavones, increased hepatic *Cyp7a1* expression and BA excretion in high-fat fed mice and in ovariectomised rats, respectively [82,83]. The bacterial isoflavone metabolite equol has also been shown to alter BA metabolism by increasing hepatic *CYP7A1* mRNA levels in the chicken embryos liver [84]. Soymilk and fermented soymilk, which contain both isoflavones, bioactive proteins, and peptides, have also been shown to attenuate hepatic cholesterol and triglycerides levels and increase hepatic *Cyp7a1* gene expression in Sprague Dawley rats [85]. It is not clear whether the effects can be attributed to either the flavonoids or the proteins/peptides or both, as soy protein alongside a high-fat diet also increased hepatic *Cyp7a1* mRNA in male Syrian Golden hamsters [86]. In addition, soy protein isolate has been shown to induce *Cyp7a1* hepatic expression and reduce hepatic cholesterol in rats [87]. The only study in humans showed no effects [81].

### 2.2. Stillbenes

The anti-atherosclerotic effects of resveratrol (3,5,4′-trihydroxy-trans-stilbene), which is found in the skin of grapes, blueberries, raspberries, and mulberries, have been widely studied in mouse models and human cell lines [88]. In mice on a high fat diet and in HepG2 cells, resveratrol increased CYP7A1 mRNA expression with activity being increased in the former [89]. Similar results were observed in HepG2 cells treated with resveratrol or resveratrol-glucuronides along with a concurrent decrease in cholesterol content and an increase in bile salt export protein (BSEP), respectively [90]. In alpha-Naphthylisothiocyanate (ANIT) induced liver injury in rats, resveratrol restored FXR and *Cyp7a1* expression and BA secretion [91]. Donryu rats implanted with an ascites hepatoma cell line and given resveratrol showed a dose-dependent reduction in serum cholesterol and excreted BAs [92]. Thus, supporting the evidence that resveratrol or its metabolites (aglycone and glucuronide), which are bioavailable in bile and plasma, 4 to 8 h after administration, may be functional bio-actives [93]. Resveratrol may confer its beneficial effects through FXR signalling targeting SIRT1, which acetylates FXR and prevents its binding with RXRα, consequently inhibiting its binding and activation of the *Cyp7a1* repressor Shp [94,95]. Resveratrol has also been shown to alter the gut microbiota profile in C57BL/6J and *ApoE-/-* mice as well as bile salt hydrolase activity and de-conjugation, thus increasing faecal excretion. This was associated with the repression of enterohepatic FXR and FGF15 signalling and increased *Cyp7a1* expression and hepatic BA synthesis [96]. In antibiotic treated mice, resveratrol has no such effects, indicating the importance of gut microbiota in resveratrol mediated effects [96].

### 2.3. Phenolic Acids and Phenolic Polypmers

Phenolic acids such as vanillic, caffeic, ferulic, and gallic acid are found in high concentrations in berries, tea, whole grains, and wine. A fermented Chinese tea, which contains a range of phenolic acids including catechin and gallic acid, has been shown to reduce LDL cholesterol in a 3-month double-blind randomised study of health and hypercholesterolemic patients, although BA levels were not measured [97,98,99]. Similarly, in hypercholesteraemic rats, an 8-week administration of the tea increased faecal BAs, but not *Cyp7a1* expression [57]. A separate study showed decreases in serum LDL and total cholesterol in hypercholesteraemic rats after 3-weeks of feeding [100]. Indeed, a phenolic acid, chlorogenic acid, found in green tea, increased in *Cyp7a1* mRNA expression in 129/Sv mice, indicating that at least some of the phenolic acid components are bioactive [101].

The beneficial effects of some phenolic polymers found in raisins, grapes, and wine (high in fibre but also tannins (proanthocyanidins)) in humans are however questionable as two human studies from the same lab showed that consuming 80–120 g raisins over a two to nine week period decreased total faecal BAs, and in another study, when tartaric acid was fed to participants, no effects on BAs were observed [102,103,104]. In contrast, 2% tannin polymers from raisins significantly increased faecal BAs and lowered LDL cholesterol in mice, although tannin monomers did not have any effect [105]. Using an in vitro study, Camire et al. proposed that the fibre in chopped raisins act as a BA sequestrant to prevent enterohepatic re-circulation, although the role of polymeric tannins cannot be ruled out as gallic acid does directly bind to taurocholic, taurodeoxycholic, and glycodeoxycholic BAs in vitro, and ellagic acid has been shown to induce genes involved in BA synthesis in mice [106,107,108]. It is therefore evident that some raisin constituents may promote BA secretion in rodents, tannin polymers (but not monomers) being among them, although their effects in humans is yet to be assessed.

### 2.4. Grape Juice, Wine and Grape Seed Extract

In-vivo and vitro effects of grapes, wine, and grape seed extract (GPSE) on BA secretion have recently been reviewed with moderate increase in *Cyp7a1* after GPSE treatments of hamsters on a 0.1% cholesterol diet while red wine procyanidins induced hepatic *Cyp7a1* in Wistar rats along with reduced LDL-C [109,110,111]. Grape juice fed to rats also increased primary BA but reduced secondary BA in the intestinal contents. Interestingly faecal counts of *Lactobacillus* and *Bifdobaceterium* were also increased [112]. In FXR knockout mice, GPSE induced faecal BA output and downregulated genes involved in intestinal BA absorption and transport. This correlated with increased *Cyp7a1* mRNA expression in the liver, decreased circulating LDL cholesterol, and decreased intestinal *Fgf15* expression [113]. In contrast, fisetin a flavonol from red wine, decreased *Cyp7a1* mRNA expression in Sprague Dawley rats, when compared to controls of mice fed a high fat diet [114]. Despite this, the rats still exhibited decreased plasma total cholesterol and LDL-cholesterol, along with decreased hepatic cholesterol content [114]. Perhaps the alternative pathway regulated by CYP27A1 may be responsible for altered BA secretion in this instance. Procyanidins may also have long-term effects; for example, in the alteration of reverse cholesterol transport in the adult offspring after intake of grape procyanidins during gestation and lactation [115]. Overall, the evidence suggests GPSE induces *Cyp7a1* mRNA expression and BA secretion into faeces. There is less evidence for wine and grape juice, although the existing publications support the same trend presented with GPSE. Once again, studies in humans are lacking.

## 3. Polyphenol Mediated Mechanisms of Action

**Figure 3:**
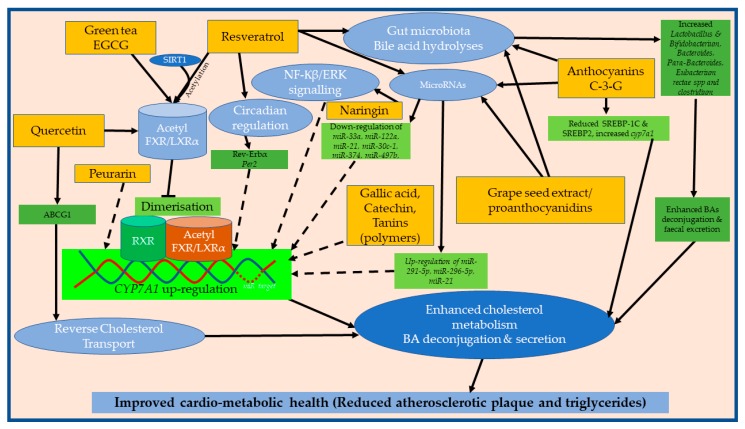
Putative mechanisms through which polyphenols modulate CYP7A1 to promote cholesterol metabolism and bile acid deconjugation, consequently attenuating atherosclerosis plaque development. Resveratrol, epigallocatechin gallate (EGCG), and quercetin increase *cyp7a1* by employing SIRT1 to acetylate FXR/LXRα; preventing its dimerisation with RXR, which then inhibits its binding and activation of CYP7A1 repressor [75,91]. Resveratrol can activate circadian proteins [116,117] and genes that subsequently upregulate *cyp7a1* and together with C-3-G, procyanidins, and naringin, can downregulate or upregulate certain microRNAs to promote CYP7A1 expression [44,118,119,120,121]. Alternatively, these may also increase bile acid hydrolases and certain gut microbiota species involved in the deconjugation and excretion of bile acids [122,123,124]. Dashed lines indicate mechanisms not completely known.

Understanding the mechanisms through which polyphenols modulate CYP7A1 to regulate cholesterol and bile acid metabolism is important and this review has highlighted that multiple pathways may be involved. These include regulation through the NF-Kβ/ERK and SIRT-RXR-FXR (LXR) signalling pathways, modification of circadian rhythm associated genes, reverse cholesterol transport, and bile salt hydrolyses (Figure 3). While there is limited experimental data on the effects of polyphenols on microRNAs and CYP7A1expression, using a database that predicts the target for microRNAs in messenger RNA in rats, several microRNAs have been recognised to target CYP7A1, and some of these microRNAs have been shown to be modulated by polyphenol supplementation (Figure 3). Polyphenols have also been shown to regulate intestinal BA transporters and alter the gut microbial composition (Figure 3) to regulate the excretion of BAs as will be discussed in Section 3.1 and Section 3.2. 

### 3.1. Control of the Apical Sodium Dependent Bile Acid Transporter (ASBT) by Polyphenols

ASBT is expressed in the ileum and the terminal portion of the small intestine, and is responsible for the re-circulation of 95% of BAs back to the liver [125]. Blocking BA enterohepatic circulation by interrupting the ASBT pathway increases hepatic BA synthesis to compensate for BAs lost in the faeces [126]. Consequently, the concentration of liver cholesterol decreases causing the activation of compensatory mechanisms to maintain cellular cholesterol homeostasis such as increased hepatic uptake of LDL cholesterol, thereby, reducing circulating cholesterol levels [126]. Therefore, ASBT is an attractive therapeutic target to lower LDL cholesterol [127]. Inhibition of ASBT by SC-435, a potent ASBT inhibitor, was found to increase feacal BA excretion, upregulate *Cyp7a1* gene expression, decrease total cholesterol, and reduce the aortic lesion area in *Apo E-/-* mice [126]. Dietary cholesterol has direct effects on ASBT function and expression [128]. In vitro, 25-hydroxycholesterol, and to a lesser extent 22-and 24-hydroxycholesterol, reduced ASBT function and mRNA levels in Caco2 cells [129]. Moreover, 25-hydroxycholesterol significantly reduced the relative activity of the human ASBT promoter in a dose-dependent manner [129]. On the other hand, 100 µM cholesterol produced no inhibitory effect on sodium-dependent taurocholate uptake into COS-7 cells that were transiently transfected with ASBT [130]. In vivo, feeding 1% cholesterol to C57BL/6J female mice for two weeks decreased ASBT mRNA abundance by 54% when compared to control, while there was no change in ASBT protein expression in Sprague Dawley rats fed with 2% cholesterol for 10 days [130,131]. It is unlikely that the differences in these two studies were due to doses given, as in the later study, a much higher dose was given, which should be more effective than a 1% cholesterol dose. Possibly, the feeding duration could have caused these differences. However, in the later study using rats, changes in other genes such as *cyp7a1* were observed, indicating that there was ample feeding time to alter gene expression. As the time of cull was not specified in the later study, circadian gene expression changes may explain the observed differences [132]. Species differences could not be ruled out either, as in the same study that used rats, 2% cholesterol significantly increased ASBT protein expression in rabbits. Although cholesterol is not a dietary bioactive, the study results are applicable as polyphenols may alter cholesterol levels, which can further alter gene expression. However, for cholesterol, the results were varied, with a trend towards an enhanced cholesterol diet leading to a decrease in the expression of ASBT. Several reports have investigated the effects of dietary bioactives on the active transport of conjugated BAs via ASBT and these are summarised in Supplementary Table S3 The evidence that polyphenols such as resveratrol alter the gut microbiota to increase faecal BAs is further explored in the following sections of the review.

### 3.2. Changes in Gut Microbiota Can Increase Bile Acid Excretion: Action of Polyphenols

Microbial metabolism and de-conjugation of BAs lead to increased diversity within the BA pool and in general, a more hydrophobic population. Increasing the hydrophobic nature of the BAs makes them less readily re-absorbed and more easily excreted into faeces. De-conjugation of BAs is catalysed by bile salt hydrolase (BSH) enzymes and genes coding for *BSH* have been detected in the main bacterial genera: *Firmicutes*, *Bacteroidetes*, and *Actinobacteria* [133]. In fact, BSH is enriched in the gut microbiota and helps the bacteria survive and colonise the gastrointestinal tract [134]. De-conjugated primary BAs that are not re-absorbed by the enterocytes enter the colon, where they are metabolised through 7-dehydroxylation into secondary BAs. 7-dehydroxylation of the primary BAs, CDCA, and CA can occur due to accessibility of the hydroxyl group, leading to DCA and LCA production. Bacteria that have 7-dehydroxylation activity are members of the *Firmicutes* phylum (*Clostridium* and *Eubacterium*) and these bacteria have a BA-inducible (BAI) gene. The bacteria are essential for the formation of secondary BAs and this is demonstrated in germ free mice, which have lower levels of secondary BAs and a smaller BA pool [135,136]. Germ-free animals accumulate cholesterol at higher levels [137], have higher levels of conjugated BAs, and significantly reduced excretion of faecal BAs [136,138].

The gut microbiota can also regulate BA metabolism by reducing the levels of tauro beta-muricholic acid (tbMCA), a naturally occurring FXR antagonist, and FXR antagonism increases *Cyp7a1* expression [135]. This has been further confirmed in a separate study, where an anti-oxidant, tempol, was used to suppress FXR signalling, which increased the levels of tbMCA [139]. Specifically, tempol decreased the genera *Lactobacillus* and *Clostridium*, which was accompanied by decreased BSH activity [139]. Oral feeding of *Lactobacillus plantarum* to mice resulted in significant reduction in LDL-C, increased faecal BA excretion, increased hepatic BA synthesis, and increased gene and protein expression of Cyp7a1 [140]. Recently, the glycine conjugate of bMCA (GbMCA) has also been found to be an FXR antagonist, which in contrast to TbMCA, was found to be resistant to faecal microbial BSH [141]. However, at present, it is not known whether TbMCA or GbMCA can antagonise FXR signalling in humans and how the human microbiota, which is more adapted to glycine conjugated BAs, metabolises GbMCA.

Human dietary interventions have shown that certain probiotics can reduce blood cholesterol by altering the microbial environment to increase BAs in the faeces [142,143]. The *Lactobacilus reuteri* strain, NCIMB 30242, which is known for its BSH activity reduced cholesterol levels in hypercholesterolaemic participants (>100) when fed an encapsulated form in a yoghurt. In a second study using the same strain of bacteria, but lyophylised, they found similar results and most importantly found that the participants had higher levels of de-conjugated BAs in their faeces [143]. In addition, live *Lactobacillus reuteri* with BSH activity when fed to pigs on a high fat, high cholesterol, low fibre diet reduced total and LDL-cholesterol concentrations [144]. Therefore, the interesting question is whether diet can alter the composition of the microbiota to decrease cholesterol levels.

Polyphenols have recently emerged as modulators of the gut microbiota [145]. Tea polyphenols have been shown to modulate the composition of the gut microbial community through the inhibition of pathogenic bacteria (*Clostridium perfringens*, *Clostridium difficile*, and *Bacteroides*) [146]. In the same study, no changes to the beneficial bacteria (*Clostridium, Bifidobacterium*, and *Lactobacillus)* were found [146], whereas, rats fed a diet containing tea polyphenols or gallotannins had reduced amounts of secondary BAs in the faeces, which suggests a reduction in bacteria with BSH activity [147]. Accordingly, in a study using rats, the supplementation of a high fat diet with catechin, curcumin, caffeic acid, rutin, ellagic acid, or quercetin reduced the concentration of secondary BAs in the faeces [148]. This suggests that the amount of de-conjugating bacteria were also reduced in the rats on these diets [148]. However, when tea flavan-3-ol monomers such as catechin were fed to humans, there was an enhanced growth of members of *Clostridium coccoides*, particularly *Eubacterium rectale*, which are known to break down BAs to secondary BAs [149]. Interestingly, *Eubacterium rectale* spp. has been shown to play a probiotic role of butyrate synthesis from carbohydrates [150,151,152] and other members of the *Clostridium coccoides* family are known to have high levels of BA 7α-dehydroxylating activity, which break down primary BAs to secondary BAs [151].

A human intervention study has shown that consumption of red wine polyphenols significantly increased the number of *Bacteroides*, *Bifidobacterium*, *Enterococcus*, *Prevotella*, *Bacteroides uniformis*, *Eggerthellalenta*, and *Blautia coccoides-E. rectale*. Correspondingly, total cholesterol was significantly decreased and most importantly, changes in cholesterol concentrations were linked to changes in the *Bifidobacteria* number, *Bifidobacteria* were probiotic, and some groups had BSH activity, although BSH activity was not measured [150]. This corresponded to a study in rats supplemented with a de-alcoholised, proanthocyanidin-rich red wine extract over 16-weeks, where the bacterial composition changed from a predominance of *Bacteroides*, *Clostridium*, and *Propionibacterium* to a prevalence of *Bacteroides*, *Lactobacillus,* and *Bifidobacterium* [153]. Interestingly, in another study, red wine tannins had no effect on the conversion of primary to secondary BAs in the faeces of rats [154].

In a human intervention study, the consumption of a wild blueberry drink, rich in polyphenols significantly increased the amount of *Bifidobacterium*, some *Bifidobacteria* are known to have BSH activity [155]. Proanthocyanidin-rich extract from grape seeds fed to healthy adults for two weeks also significantly increased the number of intestinal *Bifidobacteria* [156]. In addition, resveratrol, which is found in wine and grapes, also increased faecal counts of *Bifidobacterium* and increased *Lactobacillus* in a rat model, and in humans, it increased α-diversity such as *Barnesiella* levels that are associated with gut health and may improve cholesterol metabolism [157,158]. In one study, which substituted the water fed to rats for grape, apple, or beetroot juice, a higher concentration of primary BAs but lower concentration of secondary BAs was found in the intestinal contents along with increased amounts of cholesterol and its metabolites. Intriguingly, this corresponded to increased faecal counts of bacteria with BSH activity, *Lactobacillus* and *Bifidobacterium* [112]. Raspberry pomace containing seeds fed to rats on a high fat diet also reduced the amount of secondary BAs (LCA and DCA) in the cecum, but interestingly, the seedless fraction did not have the same effect [159]. Grape seed flour has been shown to alter the gut microbiota of male Golden Syrian hamsters. Hamsters were fed a high-fat (HF) control diet or a HF diet supplemented with 10% partially defatted grape seed flour. The Chardonnay diet altered the numbers of total bacteria and relative abundances of *Bifidobacterium*, *Lactobacillus*, and *Firmicutes* in the faeces, which were significantly lower than the control group. This was accompanied by decreased intestinal FGF15 expression and increased liver *Cyp7a1* gene expression [160]. It was suggested that alteration of the intestinal microbiota may regulate BA metabolism, but BAs were not measured in this study.

## 4. Conclusions

### 4.1. The Limitations of Existing Models

Rodent models are useful for studying the effects of polyphenols on faecal BAs, liver, and circulating cholesterol levels, but the evidence that this occurs through the regulation of CYP7A1 in humans should be examined with caution, as human cells lack a LXRα response element in the human CYP7A1 promoter. Nevertheless, the effects of polyphenols on FXR signalling are similar in mice and humans with studies showing that polyphenols affect this signalling pathway in both species [94,95]. The reverse cholesterol transport pathway and the NF-κB and ERK signalling pathway are also both inducible by polyphenols in humans and mice [69,75,161,162], providing a degree of confidence that effects observed in mouse models would also be observed in humans. Thus, indicating great potential for observations in rodent models to inform future translational studies in human. Human cell lines are the obvious next best choice for functional studies, but previous research has shown that cell density can affect the expression of *CYP7A1*mRNA [163], and it has been shown that *CYP7A1* expression is dependent on hepatocyte differentiation [164]. Specifically, cells which are more terminally differentiated are more responsive to hormones or BA conjugates when compared to less differentiated cells [13]. *CYP7A1* mRNA levels significantly increased in HepG2 cells cultured over time [14]. In addition, the presence of Fetal Bovine Serum (FBS) inhibits *CYP7A1*expression due to the presence of bovine BAs [14]. The removal of serum was shown to stimulate *CYP7A1* mRNA levels in HepG2 cells [13]. A factor in the serum such as calf BAs may be directly repressing expression of *Cyp7a1.* Alternatively, serum may influence hepatocyte differentiation, as serum-free medium has been found to promote a more differentiated phenotype than serum-containing medium [165,166]. Additionally, the presence of LDL cholesterol can affect the impact on *CYP7A1.* For example, when HepG2 cells were incubated in serum free medium with or without a red grape juice (RGJ) extract, different results were observed. In cells exposed to LDL-C, RGJ caused a marked reduction in the expression of *CYP7A1* expression, however, when LDL-C was absent, increased *CYP7A1* levels were observed on treatment with RGJ [167]. Therefore, care in the interpretation of both human and animal studies is required. Additionally, cells lines do not represent a multi-organ regulation of cholesterol and bile acid metabolism as do animal studies. Another factor that may greatly lead to disparities between experiments may be the concentrations of polyphenols used, some of which may not be physiologically relevant.

### 4.2. The Most Promising Polyphenol Candidates for Future Studies

Puerarin, an isoflavonoid, is a promising candidate for further studies. Rodent studies from three separate labs have shown that puerarin increases hepatic *Cyp7a1* expression [79,80,81]. However, when liver HepG2 cells were treated with increasing doses of puerarin, there was no effect, highlighting the disparity between human and animal studies [81]. The predominant mechanism for BA induction via puerarin in mice appears to be LXRα driven, which suggests that BA induction may not occur in human cells via the same pathway. This suggests that BA induction via polyphenols is not always LXRα driven. Quercetin has also been shown to regulate cholesterol metabolism via LXRα signalling as well as reverse cholesterol transport, which also occurs in humans, making it a promising candidate for further studies in humans [161]. There are documented mechanisms whereby polyphenols can induce CYP7A1 expression that are not driven by LXRα. For example, NF-κB and ERK signalling induces *CYP7A1*expression in human cells treated with naringin, a flavanone-7-O-glycoside [75]. In addition, attenuated FXR signalling by resveratrol, a stilbene, has been shown to induce *CYP7A1* mRNA expression in human HepG2 liver cells [89,90,95]. In conclusion, there is some promising research that polyphenols may regulate CYP7A1 expression. In addition, modification of the gut bacteria appears to be an important factor in the enhancement of BAs in the faeces, through the enrichment of bacteria with BSH activity. Red wine, rich in phenolic polymers and resveratrol, has been particularly well documented at increasing beneficial bacteria [150,153,157]. In particular, resveratrol increased levels of *Lactobacillus* and *Bifidobacterium* in mice and bacterial α-diversity in humans. These bacteria have enhanced BSH activity, which enables increased BA de-conjugation to promote faecal excretion of BAs [96]. Further studies are required to ascertain whether the parent polyphenols or their metabolites are responsible for the alterations in the gut microbiota and whether the effects seen in animal studies can be replicated in humans to inform future clinical studies.

## Figures and Tables

**Table 1:** A summary of the transcription factors and microRNAs involved in Cyp7a1 gene regulation in humans and rodents.

Factor	Humans/Human Cell Lines	Rodents	Ref.
Farnesoid X receptor, retinoic acid receptor and small heterodimer partner (FXR-RXR and SHP)	FXR is highly expressed in both the liver and ileum tissue. Ligand binding to FXR allows translocation from the cytoplasm to the nucleus to bind RXR at FXR-response elements. FXR is not able to bind to *CYP7A1* but can bind to the promoter of the *SHP* gene. SHP represses *CYP7A1* gene expression by binding to human α-fetoprotein transcription factor (FTF). FXR-RXR complex also binds illeal FGF19 (humans) or FGFR4 (mice) translocates to the liver, activates FGR4 which inhibits *c CYP7A1* transcription.	The same as humans, however, FTF is called liver receptor homolog-1 (LRH-1) in mice.	[24,25,26]
Pregnane X receptor (PXR)	PXR activation by specific bile acids such as lithocholic acid (LCA) leads to the repression of bile acid synthesis by binding and inactivating the transcription factor, hepatocyte nuclear factor 4 alpha (HNF4α) so that it can no-longer bind to its transcriptional co-activator, proliferator-activated receptor γ co-activator 1-α (PGC1α) to induce *CYP7A1* transcription.		[27]
Liver X receptor α (LXRα)	LXRα cannot bind to the human *CYP7A1* promoter due to an alteration of the DR4 motif in the BARE-I sequence. Therefore, in humans LXRα does not play a role in the regulation of CYP7A1 gene expression.	Unlike in humans, LXRα can directly bind to the *Cyp7a1* promoter to upregulate expression.	[28]
Hepatocyte nuclear factor 4 alpha/Peroxisome proliferator-activated receptor γ co-activator 1-α (HNF4α /PGC1α)	HNF4α is a transcription factor that upregulates *CYP7A1* by directly binding to its promoter along with the *trans*-activator PGC-1α.	Same as humans.	[29,30,31,32]
Peroxisome Proliferator Activated Receptor Alpha (PPARα)	In vitro PPARα over-expression in human liver cells has been shown to reduce *CYP7A1*gene expression. However, when activators of PPARα where added to non-over-expressing cells a moderate amount of inhibition was observed.	PPARα knock out mice did not show altered *Cyp7a1* levels.	[31,33]
Peroxisome Proliferator Activated Receptor gamma (PPARγ)	PPARγ activation induced *CYP7A1* expression in HepG2 cells		[34]
Forkhead box protein O1 (FoxO1)	FoxO1 is an in-direct suppressor of *CYP7A1*, there is no binding site for FoxO1 on the human *CYP7A11* promoter. FoxO1 inhibits *CYP7A1* by inhibiting expression of HNF-4α and PGC-1α TFs.	FoxO1 has the opposite function in mice and upregulates *Cyp7a1* directly by binding to its promoter.	[35,36]
Nuclear receptor subfamily 1, group D, member 1 (NR1D1 or Rev-Erba)	NR	Competes for the promoter of the clock gene *Bmal1* and mediate the circadian regulation of *Cyp7a1*	[37,38].
*Per1* and *Per2*	NR	Genetic ablation in mice disrupts normal BA control, increases serum BA and in parallel reduces *dbp* and *Cyp7a1* expression in both rats and mice	[39,40].
D site albumin promoter binding protein (DBP)	Gain-of-function studies have shown that DBP serves as a circadian activator of *CYP7A1* transcription.	*Cyp7a1* peaks after dark	[37,41,42]
Enhancer binding protein C/EBPβ-LAP	Binds cyp7a1 promoter site at DBP		[39]
miR-33		Located in the intron sequence of SREBP and regulates *Cyp7a1* expression possibly synergistically to control hepatic cholesterol metabolism and BA synthesis	[43,44,45,46]
miR-144-3p and miR-99a-3p	Target *CYP7A1* and other non-alcoholic fatty liver disease related genes		[43]
miR-122 and miR-422	Cyp7a1 also has recognition sequences for miR-122 and miR-422 in its 3′-UTR. A synthetic miR-122 mimic inhibits *CYP7A1*expression in vitro and miR-122 inhibition has shown the opposite effect		[44]
miR-24 and miR-34	Indirectly decrease *CYP7A1* by decreasing HNF4α transcription factor		[47]
miR-17	Leads a reduction in CYP7A1 mRNA expression	Reduces *Cyp7a1* mRNA expression accompanied by hepatic steatosis	[30].

## Supplementary Materials

The following are available online at https://www.mdpi.com/2072-6643/11/11/2588/s1, Figure S1: The categories and structures of dietary polyphenols. Polyphenols are subdivided into different categories: flavonoids, isoflavonoids, lignans, stilbenes, phenolic acids, and phenolic polymers. Table S1: A list of all the known BAs from rodents and humans with their abbreviations used in the review. * Dehydrocholic acid is a synthetic BA, manufactured by the oxidation of cholic acid. Table S2: The published studies that have determined whether dietary polyphenols can alter BA excretion and *Cyp7a1* expression. NR = not reported. Table S3: Effects of polyphenols on ASBT protein and gene expression. NR = not reported.

## Abbreviations

BA	bile acid
Cyp7a1	cholesterol 7 alpha-hydroxylase
RCT	reverse cholesterol transport
HDL	high-density lipoprotein
LDL	low-density lipoprotein
FXR	farnesoid X receptor
BSH	bile salt hydrolase
HF	high-fat
CDCA	chenodeoxycholic acid
