# Supplementary material for: Polyphenol Effects on Cholesterol Metabolism via Bile Acid Biosynthesis, CYP7A1: A Review
Source: Nutrients. 2019 Oct 28;11(11):2588. doi: 10.3390/nu11112588 (PMC6893479; doi:10.3390/nu11112588)
Supplement: Supplementary file 1 [file nutrients-11-02588-s001.pdf]

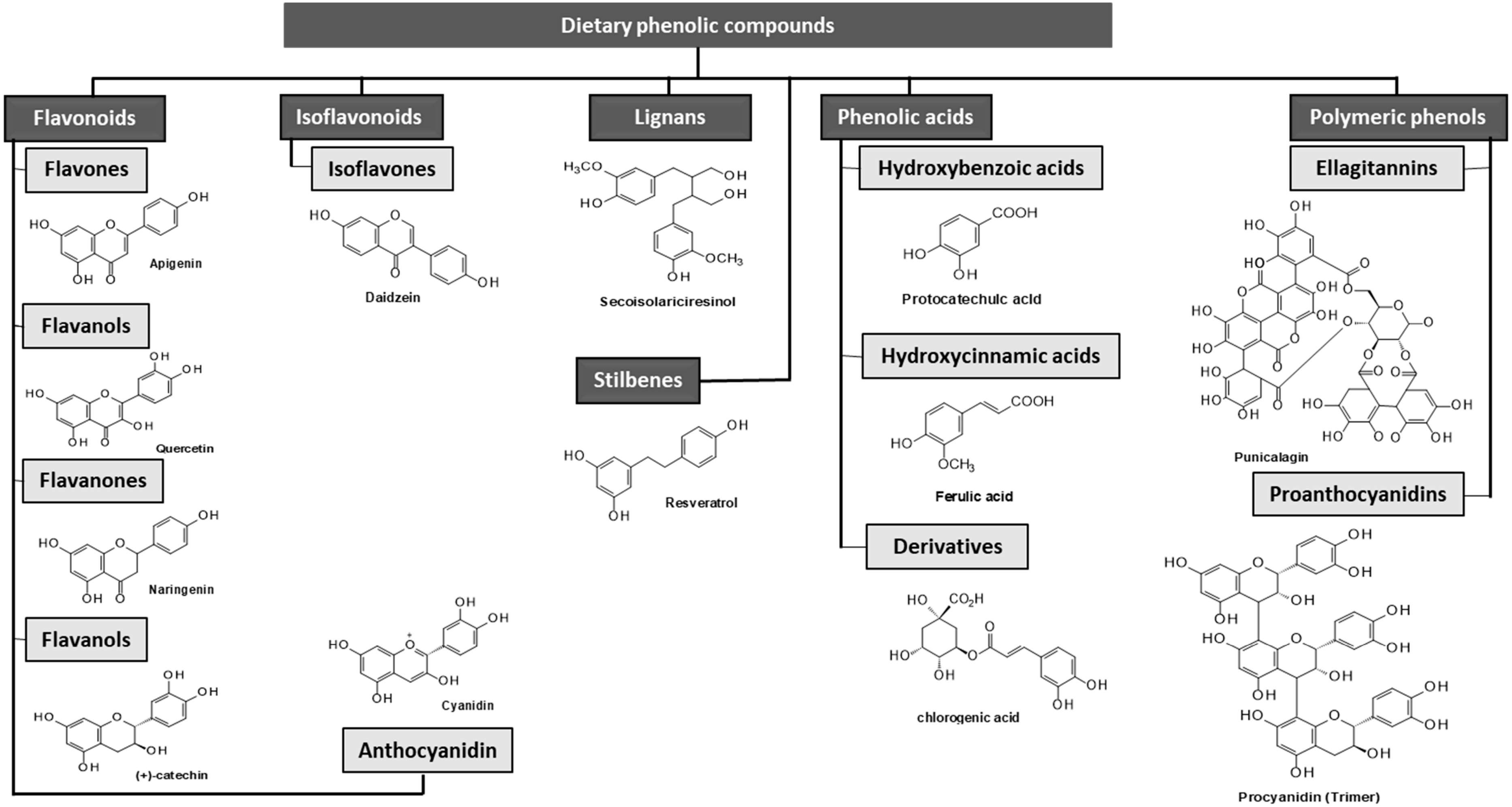

Figure S1: The categories and structures of dietary polyphenols. Polyphenols are subdivided into different categories: flavonoids, isoflavonoids, lignans, stilbenes, phenolic acids and phenolic polymers.

Table S1: A list of all the known BAs from rodents and humans with their abbreviations used in the review. *Dehydrocholic acid is a synthetic BA, manufactured by the oxidation of cholic acid.

Name	Abbrev	Type of bile acid
Chenodeoxycholic acid	CDCA	Primary
Deoxycholic acid	DCA	Secondary
Dehydrocholic acid*	DHCA	Secondary
Glycocholic acid	GCA	Conjugated primary
Glycochenodeoxycholic acid	GCDCA	Conjugated primary
Glycodeoxycholic acid	GDCA	Conjugated secondary
Lithocholic acid	LCA	Secondary
Taurocholic acid	TCA	Conjugated primary
Taurochenodeoxycholic acid	TCDCA	Conjugated primary
Taurodeoxycholic acid	TDCA	Conjugated secondary
Ursodeoxycholic acid	UDCA	Primary (mouse), Secondary (human)
Taurolithocholic acid	TLCA	Conjugated secondary
α-Muricholic acid	a-MCA	Primary (mouse), not detected (human)
β -Muricholic acid	b-MCA	Primary (mouse)
Cholic acid	CA	Primary
Glycolithocholic acid	GLCA	Conjugated secondary
Hyodeoxycholic acid	HDCA	Secondary
Muricholic acid	MCA	Primary (mouse)
Tauro-α-Muricholic acid	T-a-MCA	Conjugated primary (mouse)
Tauro- β -Muricholic acid	T-b-MCA	Conjugated primary (mouse)
Glycohyocholic acid	GHCA	Conjugated secondary
Glycoursodeoxycholic acid	GUDCA	Conjugated primary (mouse), conjugated-secondary (human)
Taurohyocholic acid	THCA	Conjugated secondary
Taurohyodeocycholic acid	THDCA	Conjugated secondary
Tauroursodeoxycholic acid	TUDCA	Conjugated-primary
Glycohyodeoxycholic acid	GHDCA	Conjugated secondary
Taurodehydrocholic acid	TDHCA	Conjugated secondary

Table S2: The published studies that have determined whether dietary polyphenols can alter BA excretion and *Cyp7a1* expression. NR= not reported

Bioactive group	Specific Bioactive	Model	Cholesterol	Hepatic *Cyp7a1* expression	Faecal bile acids	Ref
Phenolic polymers	Raisin	Human	NR	NR	Decreased	[1]
Phenolic polymers	Polymeric Tannins	Male Sprague-Dawley rats	Decreased plasma total and –LDL cholesterol	NR	Increased	[2]
Phenolic polymers	Tartaric acid	Human	NR	NR	No change decrease with raisin control.	[3]
Ellagitannin (phenolic polymer) metabolite	Ellagic acid	C3H/HeN female mice	NR	Increased mRNA	NR	[4]
Phenolic polymers	Grape seed extract (GPSE)	Golden Syrian hamsters	Decreased plasma total cholesterol	A moderate increase in mRNA and protein.	Increased levels of LCA, DCA CDCA, CA and UDCA	[5]
Phenolic polymers	GPSE	C57BL/6 WT and KO mice	Decreased serum cholesterol	Increased mRNA	Increased	[6]
Phenolic polymers	GPSE	Pigs	NR	NR	Increased	[7]
Phenolic polymers	GPSE	Wistar rats	No change in Serum cholesterol levels, but hepatic cholesterol levels decreased	No change	Decreased	[8]
Phenolic polymers	Chardonnay grape seed flour	Golden Syrian hamsters fed high-fat diets.	Decreased plasma total-, VLDL-, and LDL-cholesterol	Increased mRNA	NR	[9]
Phenolic polymers	Polyphenols in red wine	Rats	Lowered plasma LDL-cholesterol and slightly increased HDL-cholesterol.	Increased mRNA	NR	[10]
Phenolic polymers	Grape Juice (other fruit juices reported)	Rats	NR	NR	The intestinal contents contained higher concentrations of primary bile acids, but lower	[11]

					concentrations of secondary bile acids	
Phenolic polymers	GPSE	Pregnant female Wistar rats with the male adult offspring being examined	The adult male offspring had increased total cholesterol-to-HDL-C ratios.	mRNA down-regulation of Cyp7A1.	decreased	[12]
Flavonol	Fisetin	Sprague-Dawley Rats fed a high fat diet.	Elevated levels of total and LDL-cholesterol, along with hepatic cholesterol content in a high fat group were significantly reduced by fisetin.	The high fat diet Increased *Cyp7a1* mRNA, which was subsequently reversed by the fisetin	NR	[13]
Stillbenes	Resveratrol	C57BL/6 J and HepG2 cells	Improved serum lipids in mice.	Increased mRNA, protein enzyme activity (mice) and mRNA (HepG2 cells)	NR	[14]
Stillbenes	Resveratrol	Donryu rats subcutaneously implanted with an ascites hepatoma cell line of AH109A	suppressed (VLDL + LDL)-cholesterol	NR	Increased	[15]
Stillbenes	Resveratrol (15 or 30 mg/kg)	Rats with liver injury induced by alpha-Naphthylisothiocyanate (AINT).	NR	Restoration of *Cyp7a1* levels disrupted by liver injury.	Maintaining bile acid secretion originally reduced by AINT	[16]

Stillbenes	25µM Resveratrol and RSV-glucoronides	HepG2 cells	Res and Gres both lowered total cholesterol in the cells.	Significant increases in hepatic Cyp7a1 and bile salt export protein (BSEP)	NR	[17]
Flavanols	Catechins	HepG2 cells	NR	Increased *cyp7a1* mRNA after 24h. epicatechin gallate 5.5 fold; epigallocatechin-3-gallate 4.2 fold; epigallocatechin 2.9 fold and epicatechin 1.9 fold	NR	[18]
Flavanols	EGCG (40 mg kg−1 d−1 and 80 mg kg−1 d−1)	Female C57BL/6 mouse	Reduced total, and LDL cholesterol and increased HDL cholesterol.	Increased *Cyp7a1* mRNA levels	NR	[19]
Flavanols	EGCG (green tea extract)	Male C57BL mice with cholesterol-induced steatohepatitis	No changes in plasma cholesterol but a reduction in liver cholesterol in steatohepatitis mice	The mice with induced steatohepatitis demonstrated a shift from CYP7A1 pathway to the alternative acidic pathway	NR	[20]
Flavanols	EGCG	Rats	NR	increasing Cyp7a1 mRNA expression	biliary bile acid excretion was not altered, but EGCG treatment decreased bile flow by 23%	[21]
Flavanols	Lung Chen tea	Hypercholesteraemic Sprague Dawley rats	significantly lowered the serum cholesterol	*Cyp7a1* enzyme activity was not shown to be altered	Increased faecal excretion of cholesterol	[22]
Flavanols	Theaflavin-monogallates, theaflavin-3-gallate, and theaflavin-3'-	ddY mice and 5-week-old Sprague-	Black tea extract decreased the postprandial rise in blood cholesterol levels after oral administration of cholesterol in	NR	NR	[23]

	gallate (from black tea)	Dawley (SD) rats	mice and in rats the extract reduced total and LDL cholesterol.			
Flavanols	Black tea	Human study (hypercholeste rolemic)	NR	NR	No changes	[24]
Phenolic acids	Pu-erh Black tea extract 333mg/day over 3 months)	A double blind randomised study of borderline hypercholester olemic people	Reduced LDL	NR	No changes in key bile acids. Total bile acids not measured	[25]
Phenolic acid	Pu-erh Black tea (a 4-month study in 21 patients ingesting 1 g/day)	Hypercholeste rolemic patients.	Reduced cholesterol	NR	NR	[26]
Phenolic acids	8-weeks of 2% and 4% tea administration in the water	Hypercholeste raemic Sprague-Dawley rats	Lowered the serum cholesterol in the 2% and 4% group.	No change	Increase in faecal bile acids	[22]
Phenolic acids	Pu-erh black tea extract (0.3g/kg food) for 3 weeks	DdY mice fed cholesterol and hypercholester aemic Sprague-Dawley rats	Decreases in serum LDL- and total cholesterol	NR	NR	[23]
Phenolic acid	Chlorogenic acid (CGA)	male 129/sv mice	NR	Increased *Cyp7a1* mRNA	AINT increased total bile acids in serum: the effects of AINT were normalised by co-treatment with CGA.	[27]

Polyphenol rich	Pu-erh tea, theabrownin (TB) fed for 45 days	Hyperlipidemic Sprague-Dawley rats, either fed a normal diet, a high fat diet, or a high-fat diet plus TB.	LDL and total-cholesterol levels were decreased compared to the high fat diet fed group.	NR	The faecal bile acid content was higher in the TB Group than compared to both normal and high fat diet	[28]
Polyphenol rich	Glossogyne tenuifolia Cassini (herbal tea)	Syrian Hamsters	Reduced serum LDL- and total cholesterol	NR	Increased	[29]
Polyphenol rich	Ecklonia cava Brown algae extract (300 mg/kg)	C57BL6 mice fed a high fat diet	Reduced serum LDL- and total cholesterol	Increased *cyp7a1*	NR	[30]
Anthocyanin	Cyanidin-3-O-β-glucoside (anthocyanin)	Five-week-old male ApoE-/- mice and primary hepatocytes	NR	Increased *Cyp7a1* mRNA in mice and in primary hepatocytes.	Increased faecal bile acid excretion in mice and bile acid synthesis from primary hepatocytes	[31]
Anthocyanin	Anthocyanin-rich black elderberry extract (BEE) - rich in cyanidin 3-sambubioside and cyanidin 3-glucoside	Male apoE(-/-) mice fed an AIN-93M diet plus 1.25% (w/w) BEE or control diet for 6 weeks	No changes	Decreased *Cyp7a1*	NR	[32]
Anthocyanin	Anthocyanin-rich extracts from bilberry (*Vaccinium myrtillus L.),* chokeberry (*Aronia meloncarpa E.*), and grape (*Vitis vinifera*).	Fischer rats given a colon carcinogen (azoxymethane) (as the aim of this study was to assess	NR	NR	Reduction in faecal bile acids	[33]

		biomarkers of colon cancer)				
Anthocyanin	Red pericarp glutinous rice compared to brown rice (anthocyanins)	Hypercholesterolemic C57BL/6 mice (12 weeks)	Decreased serum and hepatic LDL-cholesterol	Induced *Cyp7a1* expression but decreased hepatic cholesterol levels	Not measured	[34]
Anthocyanin	Lingonberries (LBs)	Male ApoE-/- mice were fed either low-fat diet, high-fat diet, or high-fat diet with 44% lingonberries for 8 weeks.	Mice fed LBs showed reduced levels of total cholesterol compared to the HF control	Increase in *Cyp7a1* expression	NR	[35]
Anthocyanins (cyanidin 3-O-glucoside, cyanidin 3-O-rutinoside, cyanidin 3-O-galactoside, and cyanidin 3-O-galactopyranosyl-rhamnoside)	Buckwheat sprouts (*Fagopyrum esculentum Moench*)	Type 2 diabetic mice	Decreased serum cholesterol, liver cholesterol and triglycerides	Increased *Cyp7a1* mRNA	Increased faecal bile acids	[36, 37]
Flavonol	Kaempferol (150 mg/day/kg body weight)	Apo E-/-Mice	Increased HDL cholesterol and reduced total, LDL and hepatic cholesterol concentrations compared to controls	Increased hepatic mRNA expression of *Cyp7a1*	Increased faecal bile acid excretion	[38]
Flavonol	Quercetin (0.4%)	Male Wistar rats	No change in serum TC, or HDL cholesterol But, serum LDL was increased.	Elevated *Cyp7a1* mRNA and protein	Increased secreted bile acids and total liver bile acids. Upregulation of bile	[39]

					acid transporters in the liver.	
Flavonol	Black bean seed coat extract containing predominantly quercetin 3-O-glucoside and soyasaponin	C57BL/6	BBE reduced the levels of total cholesterol and HDL-cholesterol in the serum of mice fed the control diet. The effects were enhanced in high-cholesterol diet mice (decreased LDL, total cholesterol and HDL).	Increased CYP7A1 protein expression	significant increase in bile acid secretion	[40]
Flavanol combination	Quercetin and leucodelphinidin	hypercholesteraemic rats	Reduction in serum LDL- and total cholesterol, Increase in the HDL	Not observed	Increased hepatic and faecal bile acids	[41]
Flavanol combination	Quercetin fed alongside extracts of the Banyan tree, leucopelargonin and leucocyanin	Hypercholesteraemic rats	Decreased total cholesterol levels	NR	Increased hepatic and faecal bile acids	[42]
Flavanone	Naringin	Human liver HepG2 cells	NR	Induced *Cyp7a1* mRNA expression	NR	[43]
Flavone	Flavone C-glycoside rich leaf extract from Taioba (*Xanthosoma sagittifolium*)	Wistar rats	NR	NR	No change in total BAs but did diminish the levels of secondary BAs	[44, 45]
Isoflavonoid	Puerarin - the major bioactive ingredient from Japanese arrowroot (Pueraria lobate).	Male ICR mice intragastricaly administered 400mg/kg of puerarin at the same time as exposure to a reagent to	Increases in serum cholesterol and LDL were suppressed	Increases in hepatic *Cyp7a1* mRNA levels.	NR	[46]

		induce lipidemia				
Isoflavonoid	Puerarin	Spague-Dawley rats fed 300mg/kg/day puerarin and hypercholesteraemic diet	Attenuated the increased total cholesterol induced by hypercholesterolmic diet in both serum and liver	*Cyp7a1* hepatic mRNA induction	NR	[47]
Isoflavonoid	Puerarin and puerarin glycosides	C57BL/6J mice fed a 0.1% puerarin diet. Human HepG2 liver cells.	Puerarin glycosides significantly reduced plasma total cholesterol levels.	Increased hepatic *Cyp7a1* mRNA expression in the mouse study, but no effect in HepG2 cells.	NR	[48]
Isoflavones, monacolins and phytosterols.	Xuezhikang, is an extract of red yeast rice. 3 fractions containing isoflavones, monacolins or phytosterols, respectively were given to mice on a high-fat diet and compared to a control or total extract group	C57BL/6 mice	Total fraction - reduced serum TC, and LDL-C and increased serum HDL-C	The total extract and the isoflavone extract were more potent at inducing *Cyp7a1* gene expression	The total extract and all three fractions Reduced liver bile acids and increased cholesterol and bile acid secretion into the faeces.	[49]
Isoflavone	*Erythrina lysistemon*, a species of tree in the pea family, containing alpinumisoflavone	Ovariectomized rats	NR	Upregulates *Cyp7a1* liver mRNA expression	NR	[50]

Table S3: Effects of polyphenols on ASBT protein and gene expression

Food component or polyphenol	Human	Rodents	Ref
Barley	NR	Reduced ASBT gene and protein expression in the intestine by 51% and 30%, respectively in mice fed high β-glucan (fibre) barley alongside a high fat diet	[51]
EGCG	In HEK-293 cells decreased ASBT function in a time and a dose-dependent manner. Strongly inhibited ASBT activity in colonic Caco2 cells	No effects on gene expression in C57BL/6J mice	[52] [52] [53]
Procyanidin extract (GSPE)	In Caco2 cells, where GSPE reduced ASBT in a dose-dependent manner	Downregulated ASBT expression in C57BL/6 mice	[6, 54]
Resveratrol	Reduces both ASBT function and protein expression in transiently transfected COS-1 cells in a dose dependent manner, without altering gene expression. In Caco2 a high concentration of resveratrol induced ASBT gene expression	In C57BL/6J and *ApoE-/-* mice resveratrol had no effects on ASBT but increased faecal BAs. Increased levels of the genera *Lactobacillus* and *Bifidobacterium,* bile salt hydrolase (BSH) activity and thereby enhancing BA de-conjugation and faecal excretion	[55, 56]

References:

1. Spiller GA, Story JA, Lodics TA, Pollack M, Monyan S, Butterfield G, Spiller M: **Effect of sun-dried raisins on bile acid excretion, intestinal transit time, and fecal weight: a dose-response study.** *J Med Food* 2003, **6:**87-91.
2. Tebib K, Besancon P, Rouanet JM: **Dietary grape seed tannins affect lipoproteins, lipoprotein lipases and tissue lipids in rats fed hypercholesterolemic diets.** *J Nutr* 1994, **124:**2451-2457.
3. Spiller GA, Story JA, Furumoto EJ, Chezem JC, Spiller M: **Effect of tartaric acid and dietary fibre from sun-dried raisins on colonic function and on bile acid and volatile fatty acid excretion in healthy adults.** *Br J Nutr* 2003, **90:**803-807.
4. Yao R, Yasuoka A, Kamei A, Ushiama S, Kitagawa Y, Rogi T, Shibata H, Abe K, Misaka T: **Nuclear receptor-mediated alleviation of alcoholic fatty liver by polyphenols contained in alcoholic beverages.** *PLoS One* 2014, **9:**e87142.
5. Jiao R, Zhang Z, Yu H, Huang Y, Chen ZY: **Hypocholesterolemic activity of grape seed proanthocyanidin is mediated by enhancement of bile acid excretion and up-regulation of CYP7A1.** *J Nutr Biochem* 2010, **21:**1134-1139.
6. Heidker RM, Caiozzi GC, Ricketts ML: **Dietary procyanidins selectively modulate intestinal farnesoid X receptor-regulated gene expression to alter enterohepatic bile acid recirculation: elucidation of a novel mechanism to reduce triglyceridemia.** *Mol Nutr Food Res* 2016, **60:**727-736.
7. Quifer-Rada P, Choy YY, Calvert CC, Waterhouse AL, Lamuela-Raventos RM: **Use of metabolomics and lipidomics to evaluate the hypocholestreolemic effect of Proanthocyanidins from grape seed in a pig model.** *Mol Nutr Food Res* 2016, **60:**2219-2227.
8. Downing LE, Heidker RM, Caiozzi GC, Wong BS, Rodriguez K, Del Rey F, Ricketts ML: **A Grape Seed Procyanidin Extract Ameliorates Fructose-Induced Hypertriglyceridemia in Rats via Enhanced Fecal Bile Acid and Cholesterol Excretion and Inhibition of Hepatic Lipogenesis.** *PLoS One* 2015, **10:**e0140267.
9. Kim H, Bartley GE, Arvik T, Lipson R, Nah SY, Seo K, Yokoyama W: **Dietary supplementation of chardonnay grape seed flour reduces plasma cholesterol concentration, hepatic steatosis, and abdominal fat content in high-fat diet-induced obese hamsters.** *J Agric Food Chem* 2014, **62:**1919-1925.
10. Del Bas JM, Fernandez-Larrea J, Blay M, Ardevol A, Salvado MJ, Arola L, Blade C: **Grape seed procyanidins improve atherosclerotic risk index and induce liver CYP7A1 and SHP expression in healthy rats.** *Faseb j* 2005, **19:**479-481.
11. Sembries S, Dongowski G, Mehrlander K, Will F, Dietrich H: **Physiological effects of extraction juices from apple, grape, and red beet pomaces in rats.** *J Agric Food Chem* 2006, **54:**10269-10280.
12. Del Bas JM, Crescenti A, Arola-Arnal A, Oms-Oliu G, Arola L, Caimari A: **Intake of grape procyanidins during gestation and lactation impairs reverse cholesterol transport and increases atherogenic risk indexes in adult offspring.** *J Nutr Biochem* 2015, **26:**1670-1677.
13. Shin MJ, Cho Y, Moon J, Jeon HJ, Lee SM, Chung JH: **Hypocholesterolemic effect of daily fisetin supplementation in high fat fed Sprague-Dawley rats.** *Food Chem Toxicol* 2013, **57:**84-90.
14. Chen Q, Wang E, Ma L, Zhai P: **Dietary resveratrol increases the expression of hepatic 7alpha-hydroxylase and ameliorates hypercholesterolemia in high-fat fed C57BL/6J mice.** *Lipids Health Dis* 2012, **11:**56.
15. Miura D, Miura Y, Yagasaki K: **Hypolipidemic action of dietary resveratrol, a phytoalexin in grapes and red wine, in hepatoma-bearing rats.** *Life Sci* 2003, **73:**1393-1400.
16. Wang T, Zhou ZX, Sun LX, Li X, Xu ZM, Chen M, Zhao GL, Jiang ZZ, Zhang LY: **Resveratrol effectively attenuates alpha-naphthyl-isothiocyanate-induced acute cholestasis and liver

injury through choleretic and anti-inflammatory mechanisms. *Acta Pharmacol Sin* 2014, **35:**1527-1536.
17. Shao D, Wang Y, Huang Q, Shi J, Yang H, Pan Z, Jin M, Zhao H, Xu X: **Cholesterol-Lowering Effects and Mechanisms in View of Bile Acid Pathway of Resveratrol and Resveratrol Glucuronides.** *J Food Sci* 2016.
18. Lee MS, Park JY, Freake H, Kwun IS, Kim Y: **Green tea catechin enhances cholesterol 7alpha-hydroxylase gene expression in HepG2 cells.** *Br J Nutr* 2008, **99:**1182-1185.
19. Shan D, Fang Y, Ye Y, Liu J: **EGCG reducing the susceptibility to cholesterol gallstone formation through the regulation of inflammation.** *Biomedicine & Pharmacotherapy* 2008, **62:**677-683.
20. Hirsch N, Konstantinov A, Anavi S, Aronis A, Hagay Z, Madar Z, Tirosh O: **Prolonged feeding with green tea polyphenols exacerbates cholesterol-induced fatty liver disease in mice.** *Mol Nutr Food Res* 2016.
21. Hirsova P, Karlasova G, Dolezelova E, Cermanova J, Zagorova M, Kadova Z, Hroch M, Sispera L, Tomsik P, Lenicek M, et al: **Cholestatic effect of epigallocatechin gallate in rats is mediated via decreased expression of Mrp2.** *Toxicology* 2013, **303:**9-15.
22. Yang TT, Koo MW: **Chinese green tea lowers cholesterol level through an increase in fecal lipid excretion.** *Life Sci* 2000, **66:**411-423.
23. Fujita H, Yamagami T: **Extract of black tea (pu-ehr) inhibits postprandial rise in serum cholesterol in mice, and with long term use reduces serum cholesterol and low density lipoprotein levels and renal fat weight in rats.** *Phytother Res* 2008, **22:**1275-1281.
24. Mai V, Katki HA, Harmsen H, Gallaher D, Schatzkin A, Baer DJ, Clevidence B: **Effects of a controlled diet and black tea drinking on the fecal microflora composition and the fecal bile acid profile of human volunteers in a double-blinded randomized feeding study.** *J Nutr* 2004, **134:**473-478.
25. Fujita H, Yamagami T: **Antihypercholesterolemic effect of Chinese black tea extract in human subjects with borderline hypercholesterolemia.** *Nutr Res* 2008, **28:**450-456.
26. Fujita H, Yamagami T: **Efficacy and safety of Chinese black tea (Pu-Ehr) extract in healthy and hypercholesterolemic subjects.** *Ann Nutr Metab* 2008, **53:**33-42.
27. Tan Z, Luo M, Yang J, Cheng Y, Huang J, Lu C, Song D, Ye M, Dai M, Gonzalez FJ, et al: **Chlorogenic acid inhibits cholestatic liver injury induced by alpha-naphthylisothiocyanate: involvement of STAT3 and NFkappaB signalling regulation.** *J Pharm Pharmacol* 2016, **68:**1203-1213.
28. Gong J, Peng C, Chen T, Gao B, Zhou H: **Effects of theabrownin from pu-erh tea on the metabolism of serum lipids in rats: mechanism of action.** *J Food Sci* 2010, **75:**H182-189.
29. Lee YN, Hsu GS, Lin WT, Lu YF: **Hypolipidemic and Antioxidative Effects of Glossogyne tenuifolia in Hamsters Fed an Atherogenic Diet.** *J Med Food* 2016, **19:**513-517.
30. Park EY, Choi H, Yoon JY, Lee IY, Seo Y, Moon HS, Hwang JH, Jun HS: **Polyphenol-Rich Fraction of Ecklonia cava Improves Nonalcoholic Fatty Liver Disease in High Fat Diet-Fed Mice.** *Mar Drugs* 2015, **13:**6866-6883.
31. Wang D, Xia M, Gao S, Li D, Zhang Y, Jin T, Ling W: **Cyanidin-3-O-beta-glucoside upregulates hepatic cholesterol 7alpha-hydroxylase expression and reduces hypercholesterolemia in mice.** *Mol Nutr Food Res* 2012, **56:**610-621.
32. Farrell N, Norris G, Lee SG, Chun OK, Blesso CN: **Anthocyanin-rich black elderberry extract improves markers of HDL function and reduces aortic cholesterol in hyperlipidemic mice.** *Food Funct* 2015, **6:**1278-1287.
33. Lala G, Malik M, Zhao C, He J, Kwon Y, Giusti MM, Magnuson BA: **Anthocyanin-rich extracts inhibit multiple biomarkers of colon cancer in rats.** *Nutr Cancer* 2006, **54:**84-93.
34. Park Y, Park EM, Kim EH, Chung IM: **Hypocholesterolemic metabolism of dietary red pericarp glutinous rice rich in phenolic compounds in mice fed a high cholesterol diet.** *Nutr Res Pract* 2014, **8:**632-637.

35. Matziouridou C, Marungruang N, Nguyen TD, Nyman M, Fak F: **Lingonberries reduce atherosclerosis in Apoe(-/-) mice in association with altered gut microbiota composition and improved lipid profile.** *Mol Nutr Food Res* 2016, **60:**1150-1160.
36. Watanabe M, Ayugase J: **Effects of buckwheat sprouts on plasma and hepatic parameters in type 2 diabetic db/db mice.** *J Food Sci* 2010, **75:**H294-299.
37. Kim SJ, Maeda T, Sarker MZ, Takigawa S, Matsuura-Endo C, Yamauchi H, Mukasa Y, Saito K, Hashimoto N, Noda T, et al: **Identification of anthocyanins in the sprouts of buckwheat.** *J Agric Food Chem* 2007, **55:**6314-6318.
38. Hoang MH, Jia Y, Mok B, Jun HJ, Hwang KY, Lee SJ: **Kaempferol ameliorates symptoms of metabolic syndrome by regulating activities of liver X receptor-beta.** *J Nutr Biochem* 2015, **26:**868-875.
39. Zhang M, Xie Z, Gao W, Pu L, Wei J, Guo C: **Quercetin regulates hepatic cholesterol metabolism by promoting cholesterol-to-bile acid conversion and cholesterol efflux in rats.** *Nutr Res* 2016, **36:**271-279.
40. Chavez-Santoscoy RA, Gutierrez-Uribe JA, Granados O, Torre-Villalvazo I, Serna-Saldivar SO, Torres N, Palacios-González B, Tovar AR: **Flavonoids and saponins extracted from black bean (Phaseolus vulgaris L.) seed coats modulate lipid metabolism and biliary cholesterol secretion in C57BL/6 mice.** *British Journal of Nutrition* 2014, **112:**886-899.
41. Mathew BC, Yoseph BA, Dessale T, Daniel RS, Alemayehu A, Campbell IW, Augusti KT: **Hypolipidaemic effect of Leucodelphinidin derivative from Ficus bengalensis Linn on Cholesterol fed rats.** *Research Journal of Chemical Sciences* 2012, **2:**54-60.
42. Daniel RS, Devi KS, Augusti KT, Sudhakaran Nair CR: **Mechanism of action of antiatherogenic and related effects of Ficus bengalensis Linn. flavonoids in experimental animals.** *Indian J Exp Biol* 2003, **41:**296-303.
43. Liang J, Wang C, Peng J, Li W, Jin Y, Liu Q, Meng Q, Liu K, Sun H: **Naringin regulates cholesterol homeostasis and inhibits inflammation via modulating NF-kappaB and ERK signaling pathways in vitro.** *Pharmazie* 2016, **71:**101-108.
44. Picerno P, Mencherini T, Lauro MR, Barbato F, Aquino R: **Phenolic Constituents and Antioxidant Properties of Xanthosoma violaceum Leaves.** *Journal of Agricultural and Food Chemistry* 2003, **51:**6423-6428.
45. de Almeida Jackix E, Monteiro EB, Raposo HF, Vanzela EC, Amaya-Farfan J: **Taioba (Xanthosoma sagittifolium) leaves: nutrient composition and physiological effects on healthy rats.** *J Food Sci* 2013, **78:**H1929-1934.
46. Ma JQ, Ding J, Zhao H, Liu CM: **Puerarin attenuates carbon tetrachloride-induced liver oxidative stress and hyperlipidaemia in mouse by JNK/c-Jun/CYP7A1 pathway.** *Basic Clin Pharmacol Toxicol* 2014, **115:**389-395.
47. Yan LP, Chan SW, Chan AS, Chen SL, Ma XJ, Xu HX: **Puerarin decreases serum total cholesterol and enhances thoracic aorta endothelial nitric oxide synthase expression in diet-induced hypercholesterolemic rats.** *Life Sci* 2006, **79:**324-330.
48. Chung MJ, Sung N-J, Park C-S, Kweon D-K, Mantovani A, Moon T-W, Lee S-J, Park K-H: **Antioxidative and hypocholesterolemic activities of water-soluble puerarin glycosides in HepG2 cells and in C57 BL/6J mice.** *European Journal of Pharmacology* 2008, **578:**159-170.
49. Feng D, Sun JG, Sun RB, Ou-Yang BC, Yao L, Aa JY, Zhou F, Zhang JW, Zhang J, Wang GJ: **Isoflavones and phytosterols contained in Xuezhikang capsules modulate cholesterol homeostasis in high-fat diet mice.** *Acta Pharmacol Sin* 2015, **36:**1462-1472.
50. Mvondo MA, Njamen D, Kretzschmar G, Imma Bader M, Tanee Fomum S, Wandji J, Vollmer G: **Alpinumisoflavone and abyssinone V 4'-methylether derived from Erythrina lysistemon (Fabaceae) promote HDL-cholesterol synthesis and prevent cholesterol gallstone formation in ovariectomized rats.** *J Pharm Pharmacol* 2015, **67:**990-996.

51. Hoang MH, Houng SJ, Jun HJ, Lee JH, Choi JW, Kim SH, Kim YR, Lee SJ: **Barley intake induces bile acid excretion by reduced expression of intestinal ASBT and NPC1L1 in C57BL/6J mice.** *J Agric Food Chem* 2011, **59:**6798-6805.
52. Annaba F, Kumar P, Dudeja AK, Saksena S, Gill RK, Alrefai WA: **Green tea catechin EGCG inhibits ileal apical sodium bile acid transporter ASBT.** *Am J Physiol Gastrointest Liver Physiol* 2010, **298:**G467-473.
53. Liu SW, Wu ZY, Guo S, Meng XJ, Chang XD: **Polyphenol-rich extract from wild Lonicera caerulea berry reduces cholesterol accumulation by mediating the expression of hepatic miR-33 and miR-122, HMGCR, and CYP7A1 in rats.** *Journal of Functional Foods* 2018, **40:**648-658.
54. Heidker RM, Caiozzi GC, Ricketts ML: **Grape Seed Procyanidins and Cholestyramine Differentially Alter Bile Acid and Cholesterol Homeostatic Gene Expression in Mouse Intestine and Liver.** *PLoS One* 2016, **11:**e0154305.
55. Chothe PP, Swaan PW: **Resveratrol promotes degradation of the human bile acid transporter ASBT (SLC10A2).** *Biochem J* 2014, **459:**301-312.
56. Chen ML, Yi L, Zhang Y, Zhou X, Ran L, Yang J, Zhu JD, Zhang QY, Mi MT: **Resveratrol Attenuates Trimethylamine-N-Oxide (TMAO)-Induced Atherosclerosis by Regulating TMAO Synthesis and Bile Acid Metabolism via Remodeling of the Gut Microbiota.** *MBio* 2016, **7:**e02210-02215.
